# Has sentiment returned to the pre-pandemic level? A sentiment analysis using U.S. college subreddit data from 2019 to 2022

**DOI:** 10.1371/journal.pone.0299837

**Published:** 2024-03-15

**Authors:** Tian Yan, Fang Liu

**Affiliations:** Applied and Computational Mathematics and Statistics, University of Notre Dame, Notre Dame, IN, United States of America; Kitami Institute of Technology, JAPAN

## Abstract

**Background:**

As the impact of the COVID-19 pandemic winds down, both individuals and society are gradually returning to life and activities before the pandemic. This study aims to explore how people’s emotions have changed from the pre-pandemic period during the pandemic to this post-emergency period and whether the sentiment level nowadays has returned to the pre-pandemic level.

**Method:**

We collected Reddit social media data in 2019 (pre-pandemic), 2020 (peak period of the pandemic), 2021, and 2022 (late stages of the pandemic, transitioning period to the post-emergency period) from the subreddits communities in 128 universities/colleges in the U.S., and a set of school-level baseline characteristics such as location, enrollment, graduation rate, selectivity, etc. We predicted two sets of sentiments from a pre-trained Robustly Optimized BERT pre-training approach (RoBERTa) and a graph attention network (GAT) that leverages both the rich semantic information and the relational information among posted messages and then applied model stacking to obtain the final sentiment classification. After obtaining the sentiment label for each message, we employed a generalized linear mixed-effects model to estimate the temporal trend in sentiment from 2019 to 2022 and how the school-level factors may affect the sentiment.

**Results:**

Compared to the year 2019, the odds of negative sentiment in years 2020, 2021, and 2022 are 25%. 7.3%, and 6.3% higher, respectively, which are all statistically significant at the 5% significance level based on the multiplicity-adjusted p-values.

**Conclusions:**

Our study findings suggest a partial recovery in the sentiment composition (negative vs. non-negative) in the post-pandemic-emergency era. The results align with common expectations and provide a detailed quantification of how sentiments have evolved from 2019 to 2022 in the sub-population represented by the sample examined in this study.

## Introduction

### Background

While COVID-19 remains a public health priority, many governments have transitioned away from the emergency phase that gripped the globe in 2020 and 2021. With a variety of effective strategies implemented to combat the COVID-19 pandemic, including vaccination, quarantine measures, and the adoption of remote work and study routines, the impact of the pandemic on society has gradually subsided since the second half of 2021. In the U.S., nearly all state-level mask mandates had been lifted by April 2022; many educational institutions from elementary schools to higher education institutes have returned to the pre-pandemic in-person learning mode; social gatherings, conferences, sports, and entertainment events have also welcomed back participants and fans at full capacity, among others.

Despite that physical and in-person activities may have already largely recovered, the post-pandemic world does not mirror the pre-pandemic era in many aspects. Two such changes are people’s social behaviors and the public’s opinions and attitudes toward various domains and subjects and their psychological and emotional status shifts from pre-pandemic. In this work, we focus on the latter topic.

Research has been undertaken to examine sentiments and attitudes towards public health in the aftermath of various pandemic waves and post-pandemic periods, leveraging data from social media. For example, Twitter data were collected to study the sentiment distribution in India after the second wave of COVID-19 using deep neural networks [1] and the majority of sentiments were found either neutral or positive. Potential reasons behind the negative sentiment toward COVID-19 vaccine were investigated using Twitter data [2]. Tweets with negative sentiment were selected and topics implied by these tweets were discovered using topic modeling and manual thematic analysis. A subsequent study indicates that the negative sentiment towards the COVID-19 vaccine has a detrimental spillover effect on the public’s sentiment towards other vaccines such as the Measles vaccine [3]. The motivation and inclination to travel in 2021 were studied using thematic analysis, sentiment classification, and word cloud; nature-based travel was the first choice of travel after 2020 [4]. Twitter users’ sentiment change toward COVID-19 vaccination was studied [5] after the first COVID-19 vaccination was implemented in the U.S. It was found that public sentiment towards vaccination became more positive after the first dose of vaccination. Post-pandemic public opinion and sentiment on ports and corporate choice of import and export of goods were positive through frequency verification between public opinions and sentiments analysis of the influence mechanism [6].

Socioeconomic factors that may affect people’s attitude towards reopening the economy post-pandemic were studied using various data sources such as Twitter data, socioeconomic data, and COVID-19 cases [7]. It was found that people with low education levels, low income, in the labor force, and with higher residential rents were more interested in reopening the economy. Twitter data was used to learn people’s attitudes towards remote working [8] through topic modeling and deep neural networks. It found that “work-life balance”, “less stress”, “future” and “engagement” were positive topics; “virtual health”, “privacy concerns”, and “stress” were negative topics; and neutral topics included “new technologies”, “sustainability”, and “technology issues”. Sentiment analysis of Twitter-based teleworking in a post-pandemic context showed the prevalence of positive sentiments regarding telework that were generally associated with confidence, anticipation, and joy. [9]. Social media data were collected to provide a comprehensive analysis of logistics and transportation trends and showed The overall sentiment toward post-pandemic logistics in Japan was positive [10]. A study was conducted to learn public sentiment towards the government for efforts to restore the economy in Indonesia and suggested a high percentage margin between positive and negative sentiments of 37% [11].

Public sentiment toward education post-COVID-19 was also studied using Twitter data sector [12] safety was identified as a top concern for students, parents, and educators via sentiment analysis and machine learning (ML) techniques. Social media data was also used to study the attitude of the Jordanian community towards online and in-person hybrid learning [13] in the post-pandemic era. The study found that 40% of the samples displayed negative sentiments, 13% were positive, and 24.5% were neutral. A group of students at a university in the Philippines were asked about their thoughts or feelings of students [14] about the implementation of the limited face-to-face classes. The responses are dominated by positive thoughts or feelings (∼67%), as opposed to negative (∼23%) and neutral (∼10%).

There is also work that studied general sentiment change at different stages during the pandemic using other data types, such as surveys. For example, depressive symptoms were measured via the 8-item Center for Epidemiologic Studies Depression Scale in participants of ≥60 years old, which showed that the prevalence of clinically depressive symptoms was 19.8% during the pandemic, 7.2%, and 7.2% at waves 4 and 5 respectively [15]. Anxiety and depression symptoms and their recovery and loneliness in 2019 and after the first wave of COVID-19 in 2020 were studied among the general Dutch population using data from the Dutch Longitudinal Internet studies for the Social Sciences panel [16]. The study suggested the first wave of the pandemic did not negatively affect the prevalence of anxiety and depression symptoms among the general population during the first four months, but that emotional loneliness increased.

In summary, the above work used social media data to study sentiment or attitudes at a single snapshot in time in 2020, 2021, or 2022, with many focusing on data in a specific domain such as economy, education, import/export, logistics, travel, remote working, and telework. The sub-populations being studied were also diverse, including the elderly populations, college students, and the workforce, or among the general social media users, the demographics of whom are hard to define due to lack of data.

### Study objective and overview

The primary goal of this study is to examine how the sentiment shift from 2019 to 2022 and whether and when the level of negative sentiments has returned to the pre-pandemic era (2019) using college subreddit community data. As secondary objectives, we examine how other factors may affect the sentiment such as region, college type and classification, enrollment, etc.

Our study is different from the works summarized in Background. First, our study investigates general sentiment rather than sentiment in a specific domain or toward a specific topic; in addition, it examines the temporal trend of sentiment from 2019 to 2022, representing the before-pandemic baseline and several phases during the pandemic, rather than a single snapshot in time.

This study is a follow-up study to “COVID-19 sentiment analysis using college subreddit data” [17], which examined the sentiment during the early phase of the pandemic (2020) as opposed to the pre-pandemic (2019) in 8 higher-education institutes (HEI) using college subreddit data in the U.S. The current study collected subReddit data associated with 128 HEIs in the U.S., including the 8 schools studied in [17], over 4 years (August to December in 2019, 2020, 2021, and 2022, respectively), where 2021 and 2022 can be regarded as later stages of the pandemic. In other words, the scope of this study is much broader compared to [17], with a longer study period and many more schools that cover all four regions of the U.S.; the number of messages also increases from 165,570 in [17] to 3,925,509 in this study. In terms of methods, we used a pre-trained model that was an ensemble of two deep neural network models that learn sentiment from semantics, textual data, and relational data to predict sentiments of the Reddit messages collected in this study and applied a generalized linear mixture model to understand the temporal trend of sentiment from 2019 to 2022.

The remainder of the paper is structured as follows. In Data Collection, we describe the types of data collected for this study. In Methods, we introduce the ML and statistical procedures used to address the objectives of the study. The study results are presented in Results. The study limitations and future work are discussed in Discussion and the main study conclusions are presented in Conclusion.

## Materials and methods

### Data collection

First, the Reddit data collected in this study and how the data are used are in accordance with Reddit’s Terms and Conditions on data collection and usage. We also consulted the research compliance program at the University of Notre Dame (authors’ affiliation) and no Institutional Review Board (IRB) approval was needed given that the collected data were publicly accessible on Reddit and we did not collect privately identifiable data nor interact with the Reddit users. More information regarding privacy compliance is provided in S1 File.

Our study focuses on Reddit data associated with a subset of HEIs in the U.S. The inclusion/exclusion criteria for school “recruitment” into the study is that a school had an active subrededit community from 2019 to 2022 and the number of messages in a subreddit community is above some threshold. On top of that, we aim for representativeness and diversity while taking the data storage and computational cost into consideration. Specifically, we first compiled a list of HEIs with subreddits, leading to more than 400 institutions. We then dropped those schools that had a very small amount of messages in their subreddits. If a subreddit had <20 messages in each of the four years from 2019 to 2022 or <10 messages in at least two years, we dropped the school from the initial set. In addition, due to data storage and computational constraints, we further subsetted the schools, retaining the diversity and representativeness in the chosen subset, including geographical regions within the U.S. and HEI types (e.g, research universities, liberal arts colleges, and institutions specializing in particular fields such as the Naval Academy), academic ranking, level of intercollegiate athletics, among others. This eventually led to a total of 128 schools, as listed in provided in S1 File. Nevertheless, we acknowledge that the selection process, to some extent, involved subjective judgment influenced by the authors’ knowledge of the schools, despite our best efforts to maintain objectivity.

The Reddit data collection process started with the retrieval of all textual messages from the subreddits associated with each HEI in the final set of the 128 schools. We supplemented this textual corpus with additional attributes specific to each HEI, such as region, Carnegie classification of HEI (CCHEI), enrollment, graduation rate, faculty headcount, etc.

#### Reddit data collection and pre-processing

To examine the sentiment trend from 2019 and 2022, we downloaded the data from August to November, in the years 2019, 2020, 2021, and 2022 from the subreddit communities of the 128 schools. 2019 is regarded as the pre-pandemic baseline, 2020 represents the pandemic peak period, while 2021 and 2022 represent the transition to the post-emergency period. We used the Pushshift API (https://github.com/pushshift/api) to download the comment data but excluded the submission data due to its non-availability when the study was conducted.

In the downloaded message, some comments were deleted by Reddit users themselves, in which cases, “[deleted]” is used as a substitution. Comments may also be removed by Reddit moderators or administrators for various reasons, such as Reddit community rule violations, suspicious messages, spam, bots, or inappropriateness. Those comments are marked as “[removed]”. In both of the cases, we deleted them from the dataset from the subsequent sentiment analysis as they do not contain “sentiment” information. In addition. the Reddit data contains a large number of emoticons, non-standard spellings, and internet slang. We employed the RoBERTa model which is capable of handling internet slang and other non-standard spelling by using a special tokenizer, before obtaining the embeddings of the semantic information.

The messages in each school form a graph. In the graph, each node represents a message, and if one message replies to another one, they are direct neighbors and we draw a directional edge between the two. Large graphs with more than approximately 30,000 nodes would require large memories (RAM larger than 22GB), exceeding our computational ability. For that reason, we subsampled a large graph to limit its size. specifically, for those schools with over 30,000 messages, we capped it at 30,000 following the breadth-first search algorithm [18]. As suggested by its name, the algorithm explores all nodes at the present depth in a graph before moving on to the nodes at the next depth level. Specifically, we randomly selected 50 nodes to start with and then added all the neighboring nodes that were connected to each of the 50 nodes (other numbers than 50 can also be used). If there were no direct neighbors to any of the 50 nodes, we randomly selected another 50 nodes to add to the subgraph. We grew the subgraph by repeating this node-adding process sequentially until the number of the nodes in the subgraph node number got close to but did not exceed 30,000, at which point we randomly selected a set of the newly added nodes from the latest round and then randomly selected a necessary number of their neighboring nodes to make the final subgraph to contain exactly 30,000 messages.

**Fig 1:**
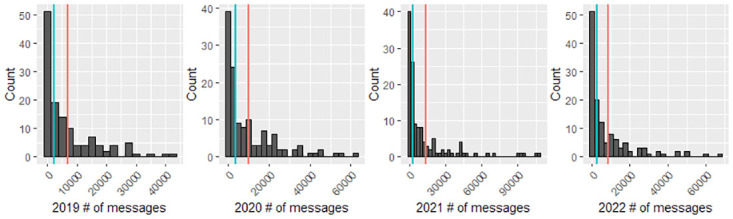
Histograms of number of messages across schools by year (blue and red lines represent median and mean, respectively).

We expect this sampling strategy will not cause notable selection bias in the sample data. There are a few reasons. First, as presented in Fig 1 in Results, there are only a small proportion of schools with more than 30,000 messages each year (2.34%, 9.38%, 14.84%, 7.03% in 2019, 2020, 2021, and 2022 respectively). In other words, all messages in most of the school subReddit communities are included in the subsequent ML and statistical analysis tasks without being subsampled. Second, nodes with connections were included in the subgroup via a largely random sample process coupled with the breadth-first search algorithm. Isolated nodes without any connection were also randomly sampled, either in the initial stage or when there were no direct neighbors to any of the already sampled nodes.

#### School-level baseline data

We consider a set of attributes associated with the schools that might impact the sentiment change from 2019 to 2022. The variables are listed in Table 1. The baseline data were collected from multiple sources—the 2020 U.S. census [19], Carnegie Classification Of Institutions Of Higher Education (CCIHE) [20], and Integrated Postsecondary Education Data System (IPEDS) [21]. For the data collected from IPEDS, if there are multiple years of data, we average them across all available years to obtain the final value for these variables. There are no missing values for this part of the collected data.

**Table 1:** Descriptive statistics of school-level baseline characteristics.

Variable	summary statistics	Source and Year
categorical variable: frequency (percentage)		
Region		- -
West	24 (18.75%)	
South	41 (32.03%)	
Northeast	31 (24.22%)	
Midwest	32 (25.00%)	
Type		CCHIE, 2021
Private	44 (34.38%)	
public	84 (65.63%)	
D1 (NCAA Division 1 school)		NCAA, 2023
Yes	108 (84.38%)	
No	20 (15.62%)	
CCHIE (Carnegie classification)		CCHIE; 2021
Baccalaureate: arts & sciences focus	7 (5.47%)	
Master’s: larger programs	1 (0.78%)	
Doctoral: high research activity	18 (14.06%)	
Doctoral: very high research activity	102 (79.69%)	
Medical (grants a medical degree?)		IPEDS; 2021
Yes	77 (60.16%)	
No	51 (39.84%)	
continuous variable: mean ± SD (min, max)		
Population (city population in 1,000)	564.4 ± 1278.8 (7.2, 8804.2)	U.S. 2020 Census
Doctoral degrees (No. of doctoral degrees granted)	315.7 ± 238.7 (0, 876)	IPEDS; 2021
Tenure (tenured/tenure-track faculty count)	1152.3 ± 682.9 (150, 3280)	IPEDS; 2019—2021
Enrollment (12-month unduplicated student enrollment in 1,000)	30.8 ± 18.1 (1.5, 101.9)	IPEDS; 2019—2021
Graduate student (12-month unduplicated graduate student enrollment in 1,000)	9.020 ± 6.153 (0, 2.920)	IPEDS; 2020—2021
Selectivity (Percent of applicants admitted)	52.5% ± 29.1% (5.0, 96.4)%	IPEDS; 2020
Graduation rate (bachelor’s within 4 yrs)	61.2% ± 20.8% (15.7, 91.3)%	IPEDS; 2019—2021

We provided descriptive statistics on these variables to examine whether the schools are balanced in these attributes. We also included these attributes in the statistical model used to estimate the sentiment trend over the 4 years. This not only helps adjust for potential confounding effects and improve the precision of parameter estimates but also allows us to examine how these factors may affect sentiment in general, aside from the pandemic.

### Methods

To analyze the data, we followed the approach in [17] by first predicting the sentiment for the collected messages using ML techniques and then estimating the sentiment trend over 2019 to 2022 using a statistical regression model. In what follows, we introduce the methods used in each step in detail.

#### Sentiment prediction

We applied the pre-trained model in [17] to predict sentiment (negative vs non-negative) for each collected message after pre-processing in this study. The model was an ensemble of a graph neural network model and a pre-trained RoBERT model. We used the pre-trained model for making predictions rather than training a prediction model based on several considerations. First, the pre-trained RoEBRTa in the ensemble was trained on a large amount of data and is widely accepted, reliable, and robust data for learning embeddings from textual data. Second, it is labor-intensive to label sentiment for messages, which is a necessary step for training a good prediction model from scratch. Third, the data used in training/testing the prediction model in [17] have commonalities with the data in this study. In fact, the whole data in the former is a subset of the data in this study; as mentioned in Introduction, this study is essentially a follow-up and scaled-up study of the study in [17] by including more schools and variables and studying a longer period. Lastly, the model trained in [17] achieved top-of-the-line accuracy performances per multiple prediction metrics, relative to the results in the literature in sentiment classification using social media data via ML techniques. All taken together, we decided to leverage the trained model in [17] to predict the sentiment in this study. We now briefly explain the components of the prediction model below, and readers may refer to [17] for more technical details as well as the numerical results of the model performance.

RoBERTa [22] is an improved version of the BERT (Bidirectional Encoder Representations from Transformers) [23] and a pretraining framework that’s based on the attention mechanism [24]. The original RoBERTa was trained on a dataset of over 160GB of uncompressed text, such as BookCorpus plus English Wikipedia (16GB), and CC-News (76GB), among others. The model we employed is based on a RoBERTa model [25] that is trained on ∼58 million messages from Twitter and fine-tuned for sentiment analysis, which is more suitable for our application. The Python code for the RoBERTa framework is adapted based on the existing work. [25] (see S1 File for the link to the code).

Embeddings for the Reddit messages were obtained from the RoBERTa model and then used in two downstream learning tasks. First, they were fed to a feed-forward neural network with softmax as the last layer to output the sentiment probabilities for the messages. Second, they were part of the input, along with the relational information among the messages, to the graph attention network (GAT) [26] to output a second set of predicted sentiment probabilities for the messages. GAT is a type of GNN model that incorporates the attention mechanism [24] to attend to neighborhoods’ features and use different weights for different nodes in a neighborhood in the graph. We treated the messages in each school as a separate directional graph in this application.

GAT and RoBERTa can be different in their sentiment prediction performance. In this particular prediction task, GAT tends to be more accurate in predicting negative sentiment and RoBERTa tends to be more accurate in predicting non-negative sentiment. To improve accuracy and obtain more robust sentiment predictions, model stacking was used in [17] to combine predicted sentiment probabilities from GAT and Roberta to output the final sentiment classification for each message.

Regarding the computational cost for running the prediction models, it took about one week to run RoBERTa and one day to run GAT, respectively, across all the messages in 128 schools on a computer with Intel(R) Xeon(R) CPU L5520 @ 2.27GHz and RAM 72.0 GB, and x64-based processor. 98.7 GB was used to store all unprocessed and processed data.

#### Statistical analysis of sentiment trend from 2019 to 2022

After having the sentiment classification for the 3,925,509 messages, we fitted a generalized linear mixed-effects model (GLMM) to examine how sentiment changes from 2019 and 2022. The GLMM is $\text{log}(\frac{\text{Pr}(y_{ik}\;\text{is}\;\text{negative})}{1-\text{Pr}(y_{ik}\;\text{is}\;\text{negative})})=\beta_{0}+\sum_{j=1}^{p}\beta_{j}x_{ijk}+z_{k}$, where the sentiment label *y*_*ik*_ (negative vs non-negative) of message *i* in school *k* is the binary response; year (categorical) and the set of variables in Table 1 are fixed-effect covariates coded in *x*_*ijk*_ for *j* = 1, …, *p* (*p* is the number of regression coefficients associated with the covariates). $z_{k}∼N\left(0,\sigma^{2}\right)$ is included as a random effect to account for the within-school dependency as the messages from the same subreddit community are likely to be correlated. Due to the different scales of magnitude across different numerical covariates *x*, standardization (shifted by sample mean and scaled by sample standard deviation) was applied to each numerical *x* before the model fitting.

From the GLMM, we estimated the odds ratios (ORs) of negative sentiment in 2020, 2021, and 2022 vs. the baseline 2019, along with 95% confidence intervals based on the Wald’s *z*-test for the ORs and p-value for testing the ORs against 1. We also estimated the effects of other school-level covariates in the model on the odds of negative sentiment in a similar manner. To correct the multiplicity issue from testing multiple odds ratios against 1 and control the overall false discovery rate (FDR), we also provided the adjusted *p*-values obtained using the FDR procedure [27].

The main assumptions for the employed GLMM include the normality of the random effect *z*_*k*_, the linearity of the covariates in the model, and no obvious outlying observations. The normality of the random effect is typical in the GLMM setting and is widely accepted and used in the statistical community. We focus on examining the main effect of the covariates in the GLMM and thus did not include any interaction terms among the covariates or higher-order terms of the numerical covariates, which also aids in result interpretation. We also calculated the Pearson residuals and plotted their histogram. The histogram of the residuals suggested the Pearson residuals were approximately normal in each sentiment category and no obvious outlying observations were detected. We included a single random variable *z*_*k*_ to account for dependency among the messages from the same subreddit in the GLMM. This implicitly assumes that the messages within the same subreddit were equally correlated. Due to a lack of data and highly unbalanced data (i.e., the number of messages per user), we did not incorporate more random effects to model other types of dependencies. For example, some messages may come from the same user, from different users in the same family, or from different users who were roommates or coworkers, etc. In other words, the dependency, if it is non-ignorable, can be complex and the data needed to account for such dependencies are largely unavailable from Reddit due to privacy concerns. In addition, even if they were available, they would be highly unbalanced across different entities, leading to potential numerical problems when running the GLMM. Since the goal of the study is to examine the temporal trend of sentiment rather than understanding different types of dependency, we believe the current model specification is sufficiently reasoned to serve the main goal. Also noted is that GLMM was applied to learn the temporal trend of sentiment over 4 years, adjusting for school-level characteristics; it was not intended for for predictive purposes, particularly given the absence of user-level attributes, except for an inclusion of a user-level random effect to account for interdependence among the data points.

## Results

### School-level characteristics

The baseline characteristics of the school-level data are summarized in Table 1. The frequency and percentage of each category are provided in the case of the categorical variables; mean, standard deviation, minimum, and maximum are provided for the continuous variables.

Fig 1 depicts the distributions of the number of messages across the 128 schools by year. The number of messages varies by school and year, but most schools have messages <30k across all 4 years. Due to computational constraints, for schools with more than 30k messages, we sampled a subgraph that has 30,000 messages (nodes) using the methods described in Reddit Data Collection and Pre-processing. This leads to a total of 3,925,509 messages in the study, the sentiments of which are predicted.

### Sentiment classification

**Fig 2:**
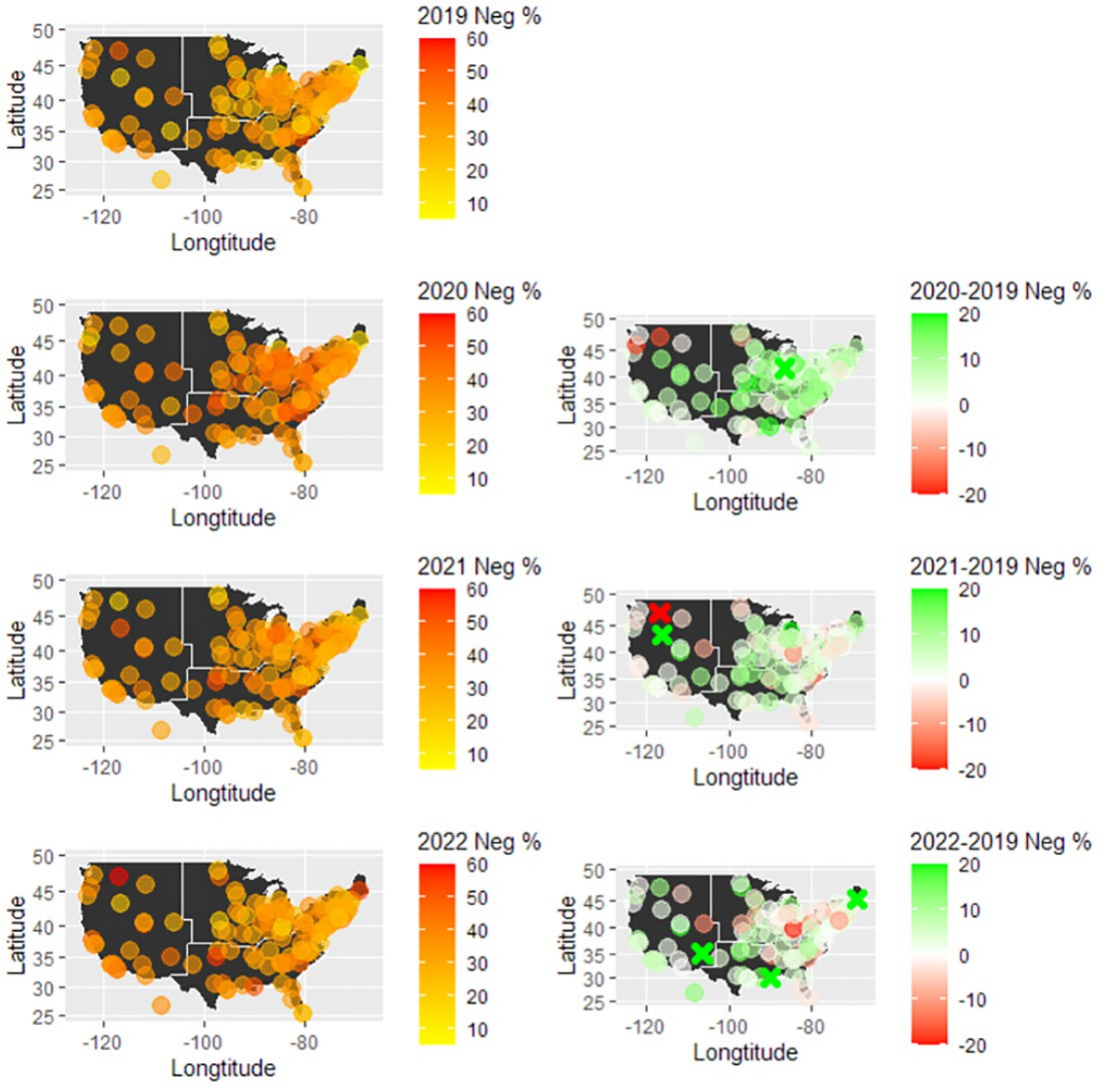
Heatmaps of negative sentiment percentage in all 128 school subreddit communities by year. Each circle represents a school. The right column shows the within-school differences between 2020 to 2022 vs. 2019 (pre-pandemic). The crosses in the 2021 and 2022 plots represent difference values outside the [−20, 20]% range. (30.38% for the University of Notre Dame in 2020; 31.25% for Boise State University, and -28.82% for the University of Idaho in 2021; and 45.72% for the University of Maine, 25.98% for the University of New Mexico-Main Campus, 31.39% for the Tulane University of Louisiana in 2022).

**Fig 3:**
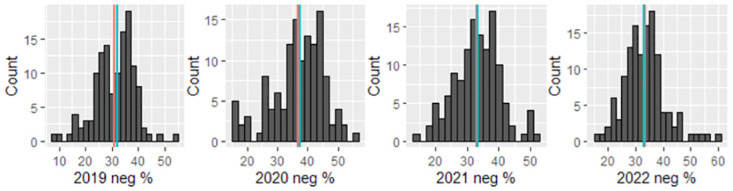
Percentage of negative sentiment distribution for all schools in each year (blue and red line represents median and mean, respectively).

We calculate the percentage of negative messages in each school year based on the predicted sentiment from the ML model and present the heatmaps (left column) in Fig 2. In the maps, each circle represents a school and its position on the map represents its geographical location in the U.S. We also plot the difference in the percentage of negative sentiment from 2020 to 2022 vs. 2019 for each school (right column) in Fig 2. Compared to the year 2019, all the other years had higher negative sentiment proportions than 2019. The year 2020 has the highest percentage of negative sentiment. For years 2021 and 2022, although the negative sentiment proportions are still higher than in 2019, they are lower than in 2020. Similar trends can be observed in Fig 3 that depicts the distribution of negative sentiment percentage across the schools by year.

### Temporal sentiment trend and school-level covariate effect

**Fig 4:**
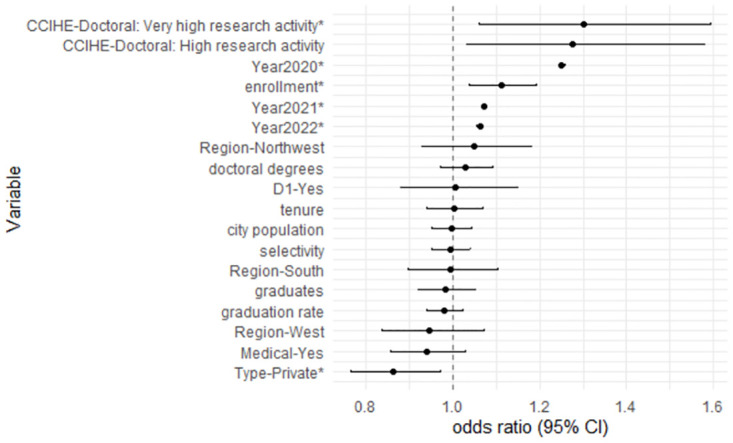
Forrest plot of estimated odds ratios of negative sentiment for the model covariates (vs. its reference level or with 1SD increase) in the GLMM with 95% confidence intervals. An asterisk * indicates a statistically significant effect per the adjusted p-value (Table 2).

The GLMM model was run on complete records only (a total of 3,925,509 messages). There is a significant imbalance in the factor CCHIE, with only one school classified as “Master’s Colleges & Universities: Larger Programs” and seven schools as “Baccalaureate: arts & sciences focus”, which could lead to potential computational and inferential problems in the GLMM setting. We thus combined the two categories as one and referred to it as “Baccalaureate/Master’s Colleges/Universities”. We used the glmer function in R package lme4 to run the GLMM and the p.adjust function in R package stats to obtain FDR adjusted *p*-values in testing whether the OR associated with a covariate in the GLMM is one or not. The inferential results are presented in Table 2 and Fig 4.

**Table 2:** Estimated effects of covariates on the odds of negative sentiment.

Covariate		odds ratio (95% CI)	*p*-value	
			raw	adjusted^†^
Region	Midwest	-	-	-
	Northwest	1.048 (0.930, 1.182)	0.442	0.646
	South	0.995 (0.898, 1.103)	0.925	0.936
	West	0.947 (0.836, 1.072)	0.389	0.616
Type	Public	-	-	-
	Private	0.864 (0.767,0.972)	0.015	0.042
Year	2019	-	-	-
	**2020**	**1.250 (1.243, 1.258)**	**<0.001**	**<0.001**
	**2021**	**1.073 (1.066, 1.079)**	**<0.001**	**<0.001**
	**2022**	**1.063 (1.056, 1.069)**	**<0.001**	**<0.001**
D1	No	-	-	-
	Yes	1.007 (0.88,1.151)	0.923	0.936
CCHIE	Baccalaureate or Master’s	-	-	-
	Doctoral: high research activity	1.276 (1.031, 1.58)	0.025	0.059
	**Doctoral: very high research activity**	**1.301 (1.062, 1.593)**	**0.011**	**0.035**
Medical	No	-	-	-
	Yes	0.940 (0.858,1.029)	0.180	0.380
city population^‡^		0.997 (0.953, 1.042)	0.878	0.936
**enrollment** ^‡^		**1.113 (1.038, 1.193)**	**0.003**	**0.010**
doctoral degrees^‡^		1.030 (0.972, 1.093)	0.318	0.604
tenure^‡^		1.003 (0.941, 1.069)	0.936	0.936
graduate student^‡^		0.984 (0.921, 1.052)	0.641	0.870
selectivity^‡^		0.996 (0.953, 1.042)	0.869	0.936
graduation rate^‡^		0.981 (0.941, 1.023)	0.375	0.616

^†^ The multiplicity-corrected/adjusted p-values were calculated using the FDR procedure [27].

^‡^ For numerical variables, the presented OR is associated with one SD increase.

The rows with—as entries are the reference categories for the categorical covariates. The **bold** rows are the covariates/levels that are statistically significant if <0.05 is used for the adjusted p-values.

We use adjusted p-value <0.05 as the cutoff for a statistically significant effect based on the adjusted p-value. Year has a statistically significant effect on Sentiment. The odds of having negative sentiments in 2020, 2021, and 2022, are 25.0%, 7.3%, and 6.3% higher, respectively, than that in 2019, suggesting the likelihood of negative sentiment increased significantly during the pandemic compared to before the pandemic. The negative sentiment proportion in the second half of 2021 almost returned to the pre-pandemic level and further decreased slightly in the second half of 2022. Overall, we may conclude the level of negative sentiment has decreased as the pandemic gradually subsided and everyday life resumed its usual course, although it might remain elevated in 2022 compared to the same period before the pandemic.

Among the other covariates examined in the GLMM, Enrollment is statistically significantly associated with sentiment. For every one SD (16,551) increase in enrollment, the odds of having negative sentiment go up by 11.3%, implying larger enrollment tends to have a negative impact on Sentiment. Compared to Master’s/Baccalaureate universities/colleges, the odds of having negative sentiment is 30.1% higher (adjusted p-value = 0.035) in doctoral schools with very high research activity and 27.6% (adjusted p-value = 0.059) in doctoral schools with high research activity. The observations exhibit rational comprehensibility; there is constant pressure on both students and faculty members in HEIs with the requirement of research productivity and excellence, which is likely linked with the higher negative sentiment in those schools and the communities that are associated with them. Private schools tend to have lower odds of negative sentiment (13.6% lower, adjusted p-value = 0.042) than public schools. The rest of the examined covariates do not have a statistically pronounced effect on sentiment, such as region, D1 school or not, a medical school or not, selectivity, etc.

The CIs associated with the ORs of the year comparisons are much narrower compared to those associated with other factors. This is because Year is the only within-cluster factor whereas the others are between-cluster factors, where cluster here refers to subreddit community or school in the GLMM. The variance of the effect of a between-cluster factor contains both the within-cluster sampling variability and the between-cluster variance (variance across clusters) whereas that for a within-factor factor only contains the former and is thus smaller. The precise estimates for the ORs in the year comparison benefit from the huge number of messages, where the precision on the estimated effects of the between-school factors is more determined by the number of schools, which is 128.

## Discussion

We collected subReddit data from 128 college communities on Reddit in the U.S. and some school-level baseline covariates to study sentiment change from 2019 to 2022 that covered the pre-pandemic period to several stages of the COVID-19 pandemic.

To our knowledge, this is the first study to examine the temporal trend of general sentiment from the pre-pandemic (2019) to a near-post-pandemic period (2nd half of 2022). While there exist studies that examine sentiments related to the pandemic (see Introduction), they often focus on a single snapshot in time during the pandemic or on a highly specific domain or topic in the post-pandemic period. While there are no existing studies for a direct side-by-side comparison with our results, the overall trend in mental recovery post-2020, as indicated by our study, aligns with findings in positive attitudes toward different topics in several studies conducted in 2022 and 2023 (e.g., [1, 8–11, 14]).

Since we used Reddit data, only schools with active subreddits from 2019 to 2022 are eligible, which are the schools that are relatively well-known and have active online communities on social media. The Reddit users who post in those schools’ subreddit are not restricted to students, professors, or staff members who are affiliated with the schools, and they may also include those who might be interested in the subreddit (e.g. those who live in the city as a school, alumni, family members, and friends of those who are affiliated with the schools, fans of the athletic programs of the schools, etc). While the study results can be generalized to the sub-population the collected data represents and reflects the sentiment changes from 2022 to 2019 in that subpopulation, they would not be immediately generalized to the general population without understanding the demographics of the users (which we don’t have data on).

We opted to employ a pre-trained sentiment classification model [17]. Although the model boasts considerable predictive capabilities and there is some overlap between the training/testing data used for that model and the data utilized in the present study, the data in this study are much more comprehensive. The classification model performance may be further improved if it can be fine-tuned using more training data in this study; an interesting topic to be explored in future work.

A reviewer pointed out that the binary classification of sentiment used in the ML community is blunt as sentiment can have many subtypes. While we agree, we chose to use binary classification out of several considerations. First, while a binary classification is not as granular, it is less subject to bias and subjectivity and is thus more robust and generalizable compared to a classification scheme with more subtypes. In addition, training an ML model to predict a categorical outcome with more levels would be more difficult and require a decent amount of training data in each level of the outcome and a larger model with more parameters. For example, Yan and Liu [17] trained ML models to predict 3-category outcomes (positive, negative, neutral) and 5-category outcomes (very positive, positive, negative, very negative, neutral), the classification accuracy on sentiments dropped to ∼60% for the former and ∼50% for the latter from ∼85% using 2-category outcomes. Third, a binary categorization is sufficient for achieving the main goal of this study. Taken together, we have chosen to use the negative vs. non-negative sentiment classification in this study. That said, we will continue to work on developing innovative methods and ML approaches aimed at enhancing the classification of sentiment subtypes, collaborating with subject matter experts.

Since the collected subreddit data do not contain individual-level demographic information about the users who posted the messages, which can be highly sensitive and pose privacy risks for re-identification, the covariates examined in the GLMM include only school-level public information. The current GLMM does not examine time-varying covariates except for the year itself. A potential interesting extension to the current study is to include time-varying covariates, such as the state-level vaccination percentage, unemployment rate, and inflation rate, in the model. On the other hand, including those time-varying covariates may blunt the temporal signal as observed in the current data due to their correlation with year. Regardless, considering these variables can help interpret the temporal trend of the sentiment qualitatively. For example, the significant drop in negative sentiment in 2021 compared to 2020 may likely be due to the availability of COVID vaccines and more effective treatments for COVID-19, giving people hope and a positive outlook that things would return to normal. Though the negative sentiment level in 2022 is still lower than in 2020, This could be attributed to the possibility that it takes more time for individuals to achieve full mental recovery. Additionally, certain factors might contribute to negative sentiment even in the absence of the pandemic. Examples include the substantial layoffs in major tech companies in 2022, impacting college and university students, as well as the significant rise in living costs during the same period. Nevertheless, these are conjectures lacking formal causality analysis and primarily serve as hypotheses that require validation through a rigorous study using a well-defined dataset, designed to understand the underlying reasons behind the observed emotional shift.

## Conclusion

We collected subreddit messages associated with 128 HEIs in the U.S., covering the pre-pandemic period (2019) and various stages of the pandemic (2020, 2021, and 2022) to understand how sentiment evolved from 2019 to 2022. The results suggest a notable recovery in the sentiment composition (negative vs. non-negative) in 2022 and 2011 with a drop of 18% to 19% in the odds of negative sentiment, respectively, from 2020, indicating a positive shift in the overall sentiment landscape compared to the peak period of the pandemic. Compared to the pre-pandemic era (2019), the odds of negative sentiment were still 6% to 7% higher in 2021 and 2022, a phenomenon possibly influenced by factors such as prolonged mental recovery from the pandemic, external events like tech company layoffs, and increased living costs in 2022.

The study’s insights could have implications for policymakers, educational institutions, and mental health practitioners, providing them with a better and more nuanced understanding of the lingering effects of the pandemic on sentiment within the higher education community. Additionally, the methodology employed in this research, combining ML and statistical analysis, contributes to the methodological toolkit for sentiment analysis in large-scale social media datasets.

## Supporting information

S1 File The file contains more information about the data and privacy compliance, the code, and the list of HEIs included in this study. (ZIP)

## References

[1] Shopnil MSI, Hasan SM, Srizon MAY, Faruk MF. Post-Pandemic Sentiment Analysis Based on Twitter Data Using Deep Learning. In: 2022 25th International Conference on Computer and Information Technology (ICCIT). IEEE; 2022. p. 704–709.

[2] Ng QX, Lim SR, Yau CE, Liew TM. Examining the prevailing negative sentiments related to COVID-19 vaccination: Unsupervised deep learning of Twitter posts over a 16-month period. Vaccines. 2022;10(9):1457. doi: 10.3390/vaccines10091457 36146535 PMC9503543

[3] Ng QX, Teo YQJ, Kiew CY, Lim BPY, Lim YL, Liew TM. Examining the Prevailing Negative Sentiments Surrounding Measles Vaccination: Unsupervised Deep Learning of Twitter Posts from 2017 to 2022. Cyberpsychology, Behavior, and Social Networking. 2023;. 10.1089/cyber.2023.0025 37358808

[4] Bhalla R, Chowdhary N, Ranjan A. Spiritual tourism for psychotherapeutic healing post COVID-19. Journal of Travel & Tourism Marketing. 2021;38(8):769–781. doi: 10.1080/10548408.2021.1930630

[5] Bustos V, Comer C, Manstein S, Laikhter E, Shiah E, Xun H, et al. Twitter voices: Twitter users’ sentiments and emotions about COVID-19 vaccination within the United States. Eur J Environ Public Health. 2022;6(1):em0096. doi: 10.21601/ejeph/11499

[6] Yi K, Li Y, Chen J, Yu M, Li X. Appeal of word of mouth: influences of public opinions and sentiment on ports in corporate choice of import and export trade in the post-COVID-19 era. Ocean & Coastal Management. 2022;225:106239. doi: 10.1016/j.ocecoaman.2022.106239 36467315 PMC9700815

[7] Rahman MM, Ali G, Li XJ, Paul KC, Chong PH. Twitter and census data analytics to explore socioeconomic factors for post-COVID-19 reopening sentiment. arXiv preprint arXiv:200700054. 2020;.

[8] Saura JR, Ribeiro-Soriano D, Saldana PZ. Exploring the challenges of remote work on Twitter users’ sentiments: From digital technology development to a post-pandemic era. Journal of Business Research. 2022;142:242–254. doi: 10.1016/j.jbusres.2021.12.052

[9] Rojas Rincón JS, Riveros Tarazona AR, Mejía Martínez AM, Acosta-Prado JC. Sentiment Analysis on Twitter-Based Teleworking in a Post-Pandemic COVID-19 Context. Social Sciences. 2023;12(11):623. doi: 10.3390/socsci12110623

[10] Hirata E, Matsuda T. Examining logistics developments in post-pandemic Japan through sentiment analysis of Twitter data. Asian Transport Studies. 2023;9:100110. doi: 10.1016/j.eastsj.2023.100110

[11] Erfina A, Fitina L, Hartanto P, Saepudin S, Rahmalenia D, Maulinda D, et al. Indonesia’s Economic Recovery Post Covid-19 Pandemic Sentiment Analysis. In: 2022 IEEE 8th International Conference on Computing, Engineering and Design (ICCED). IEEE; 2022. p. 1–4.

[12] Ismail H, Khalil A, Hussein N, Elabyad R. Triggers and Tweets: Implicit Aspect-Based Sentiment and Emotion Analysis of Community Chatter Relevant to Education Post-COVID-19. Big Data and Cognitive Computing. 2022;6(3):99. doi: 10.3390/bdcc6030099

[13] Qaqish E, Aranki A, Etaiwi W. Sentiment analysis and emotion detection of post-COVID educational Tweets: Jordan case. Social Network Analysis and Mining. 2023;13(1):39. doi: 10.1007/s13278-023-01041-8 36880094 PMC9977637

[14] Cahapin EL, Santiago CS Jr, Malabag BA, Reyes JL, Legaspi GS, Benedicto MJ, et al. Sentiment Analysis of Students’ Perception Towards the Implementation of Limited In-Person Learning: A Post-Pandemic Perspective. International Journal of Computing Sciences Research. 2023;7:1664–1684. doi: 10.25147/ijcsr.2017.001.1.126

[15] Briggs R, McDowell CP, De Looze C, Kenny RA, Ward M. Depressive symptoms among older adults pre–and post–COVID-19 pandemic. Journal of the American Medical Directors Association. 2021;22(11):2251–2257. doi: 10.1016/j.jamda.2021.09.003 34597531 PMC8436876

[16] Van der Velden PG, Hyland P, Contino C, von Gaudecker HM, Muffels R, Das M. Anxiety and depression symptoms, the recovery from symptoms, and loneliness before and after the COVID-19 outbreak among the general population: Findings from a Dutch population-based longitudinal study. PloS one. 2021;16(1):e0245057. doi: 10.1371/journal.pone.0245057 33411843 PMC7790276

[17] Yan T, Liu F. COVID-19 sentiment analysis using college subreddit data. PLoS One. 2022;17(11):e0275862. doi: 10.1371/journal.pone.0275862 36331928 PMC9635711

[18] Moore EF. The shortest path through a maze. In: Proc. of the International Symposium on the Theory of Switching. Harvard University Press; 1959. p. 285–292.

[19] 2020 Census Results. 2020 Census Results; 2023. https://www.census.gov/programs-surveys/decennial-census/decade/2020/2020-census-results.html.

[20] Indiana University Center for Postsecondary Research. Carnegie Classifications 2021 public data file; 2022. http://carnegieclassifications.acenet.edu/downloads/.

[21] Integrated Postsecondary Education Data System. Integrated Postsecondary Education Data System; 2023. https://nces.ed.gov/ipeds.

[22] Liu Y, Ott M, Goyal N, Du J, Joshi M, Chen D, et al. Roberta: A robustly optimized bert pretraining approach. arXiv preprint arXiv:190711692. 2019;.

[23] Devlin J, Chang MW, Lee K, Toutanova K. Bert: Pre-training of deep bidirectional transformers for language understanding. arXiv preprint arXiv:181004805. 2018;.

[24] Vaswani A, Shazeer N, Parmar N, Uszkoreit J, Jones L, Gomez AN, et al. Attention is all you need. Advances in neural information processing systems. 2017;30.

[25] Barbieri F, Camacho-Collados J, Neves L, Espinosa-Anke L. Tweeteval: Unified benchmark and comparative evaluation for tweet classification. arXiv preprint arXiv:201012421. 2020;.

[26] Veličković P, Cucurull G, Casanova A, Romero A, Lio P, Bengio Y. Graph attention networks. arXiv preprint arXiv:171010903. 2017;.

[27] Benjamini Y, Hochberg Y. Controlling the false discovery rate: a practical and powerful approach to multiple testing. Journal of the Royal Statistical Society: Series B (Methodological). 1995;57(1):289–300.

