# Supplementary material for: Has sentiment returned to the pre-pandemic level? A sentiment analysis using U.S. college subreddit data from 2019 to 2022
Source: PLoS One. 2024 Mar 15;19(3):e0299837. doi: 10.1371/journal.pone.0299837 (PMC10942064; doi:10.1371/journal.pone.0299837)
Supplement: S1 File — (ZIP) [file pone.0299837.s001.zip › Post_Covid_19_Sentiment_Analysis_with_Graph_Neural_Network.pdf]

Has Sentiment Returned to the Pre-pandemic Level? A Sentiment Analysis Using U.S. College Subreddit Data from 2019 to 2022

Tian Yan, Fang Liu*

Applied and Computational Mathematics and Statistics, University of Notre Dame, Notre Dame, IN 46556, United States

* fliu2@nd.edu

S1 File. The file contains more information about the data and privacy compliance, the code, and the list of HEIs included in this study.

Data The data we collected from Reddit for this study include 1) userID, 2) time of a posted message, 3) content of a message, 4) dyadic relation between two messages in terms of whether one is a reply to the other. There is no information in the collected data that allows direct identification of a user in the data. The information in 'userID' was not used in our model training or methodological development and was replaced by dummy integers after the data were downloaded.

The data collection complies with the Reddit privacy policy [1], Reddit user agreement [2], and Reddit API's term of use [3]. The data are anonymized in the sense that we replaced the actual Reddit userIDs in the data with dummy IDs (integers 1, 2, ...), further limiting potential privacy risk (e.g., through record linkage) without losing information for learning tasks based on the data. The data are available at `https://www.kaggle.com/datasets/avaytt/reddit-messages-from-selected-universities/data`.

The research compliance program at the University of Notre Dame (authors' affiliation) confirmed no Institutional Review Board (IRB) approval was needed given that the collected data were publicly accessible on Reddit and we did not collect privately identifiable data nor interacted with the Reddit users.

Code The code for this study can be download from `https://github.com/AlvaYan/postCOVIDSentiAnalysis`). The Python code for the RoBERTa framework that we applied is adapted from [4] and is available at `https://huggingface.co/cardiffnlp/twitter-roberta-base-sentiment`; the Python code for training the GAT NN is adapted from [5] (`https://github.com/Jhy1993/HAN`).

List of HEIs included in this study (alphabetically) Amherst College, Arizona State University Campus Immersion, Binghamton University, Boise State University, Boston College, Boston University, Bowdoin College, Bowling Green State University-Main Campus, Brigham Young University, Brown University, California Institute Of Technology, California State University-Fullerton, California State University-Northridge, Carleton College, Carnegie Mellon University, Case Western

Reserve University, Central Michigan University, Clemson University, Colorado State University-Fort Collins, Columbia University in the City of New York, Cornell University, Dartmouth College, Depaul University, Drexel University, Duke University, Emory University, Florida International University, Florida State University, George Mason University, George Washington University, Georgetown University, Georgia Institute of Technology-Main Campus, Georgia State University, Grinnell College, Harvard University, Illinois Institute of Technology, Indiana University-Bloomington, Iowa State University, Johns Hopkins University, Kansas State University, Marquette University, Michigan State University, Montana State University, New Jersey Institute Of Technology, New York University, North Carolina State University at Raleigh, North Dakota State University-Main Campus, Northwestern University, Ohio State University-Main Campus, Oklahoma State University-Main Campus, Oregon State University, Pomona College, Princeton University, Purdue University-Main Campus, Reed College, Rensselaer Polytechnic Institute, Rice University, Rutgers University-New Brunswick, San Diego State University, Stanford University, Stony Brook University, SUNY at Albany, Syracuse University, Temple University, Texas A & M University-College Station, Texas Tech University, The College of William and Mary, The Pennsylvania State University, The University of Texas at Austin, Tulane University of Louisiana, United States Naval Academy, University at Buffalo, University of Alabama at Birmingham, University of Arizona, University of Arkansas, University of California-Berkeley, University of California-Los Angeles, University of California-San Diego, University of California-Santa Cruz, University of Chicago, University of Cincinnati-Main Campus, University of Dayton, University of Florida, University of Georgia, University of Hawaii at Manoa, University of Houston, University of Idaho, University of Illinois Chicago, University of Illinois Urbana-Champaign, University of Iowa, University of Kansas, University of Kentucky, University of Louisiana at Lafayette, University of Maine, University of Maryland-College Park, University of Massachusetts-Amherst, University of Memphis, University of Miami, University of Michigan-Ann Arbor, University of Minnesota-Twin Cities, University of Mississippi, University of Missouri-Columbia, University of Nebraska-Lincoln, University of Nevada-Las Vegas, University of New Hampshire-Main Campus, University of New Mexico-Main Campus, University of North Carolina at Chapel Hill, University of North Carolina at Charlotte, University of North Dakota, University of Notre Dame, University of Oklahoma-Norman Campus, University of Pennsylvania, University of Pittsburgh-Pittsburgh Campus, University of South Carolina-Columbia, University of South Florida, University of Utah, University of Vermont, University of Virginia-Main Campus, University of Washington-Seattle Campus, University of Wisconsin-Madison, Vanderbilt University, Villanova University, Virginia Tech, Wake Forest University, Washington University in St Louis, West Virginia University, Western Michigan University, Yale University.

References

1. Reddit Privacy Policy. `https://www.redditinc.com/policies/privacy-policy`.
2. Reddit User Agreement. `https://www.redditinc.com/policies/user-agreement-september-12-2021`.

3. Reddit API Terms of Use.
https://www.redditinc.com/policies/data-api-terms.

4. Barbieri F, Camacho-Collados J, Neves L, Espinosa-Anke L. Tweeteval: Unified benchmark and comparative evaluation for tweet classification. arXiv:201012421. 2020;.

5. Wang X, Ji H, Shi C, Wang B, Ye Y, Cui P, et al. Heterogeneous graph attention network. In: The World Wide Web Conference; 2019. p. 2022–2032.
